# Supplementary material for: Pre-Operative MDCT Staging Predicts Mesopancreatic Fat Infiltration—A Novel Marker for Neoadjuvant Treatment?
Source: Cancers (Basel). 2021 Aug 28;13(17):4361. doi: 10.3390/cancers13174361 (PMC8430607; doi:10.3390/cancers13174361)

Figure S1. Flow chart representing patient selection for study inclusion (CRM: Circumferential resection margin; hPDAC: Ductal adenocarcinoma of the pancreatic head; PV: Portal vein; SMA: Superior mesenteric artery).

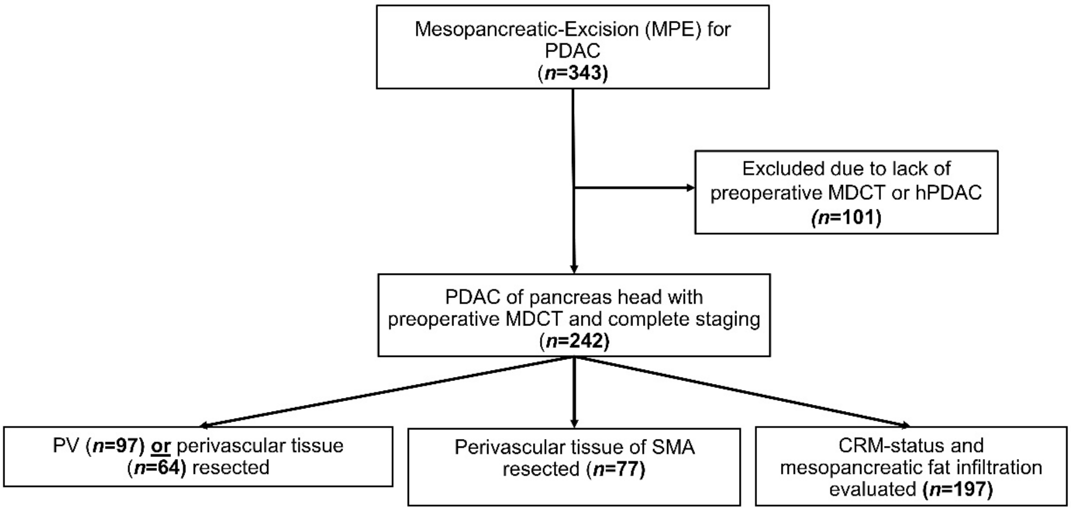

Supplement: Supplementary file 1 [file cancers-13-04361-s001.zip › cancers-1359644-supplementary.pdf]
